# Auditory object feature maps with a hierarchical network of independent components?

**DOI:** 10.1186/1471-2202-15-S1-P66

**Published:** 2014-07-21

**Authors:** Jean Rouat, Simon Brodeur, Éric Plourde

**Affiliations:** 1 NECOTIS, Département génie électrique, génie informatique, Université de Sherbrooke, Québec, Canada, J1K 2R1

## 

**Figure 1:**
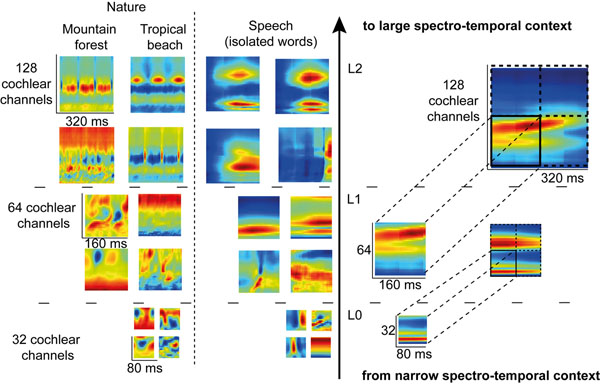
Learned representations illustrated for speech and natural sounds. FastICA training is first done on patches of 80ms x 32 cochlear channels of the envelopes coming from a 128 channels cochleagram (Level L0). Then, patches of 160ms x 64 cochlear channels are created at level L1 with a concatenation through time and space of the learned L0 features. FastICA is then performed on these larger patches to generate the new L1 representations. The same procedure is repeated for level L2.

Auditory Object representation in the brain is still a controversial question [1,2]. Kumar *et al. *[3] discuss the hierarchical organization for auditory object perception and observe that the Planum Temporale (PT) area of the cortex encodes invariant representations of the spectral envelops of sounds. Many other studies find maps of representations elsewhere in the brain (Cochlear Nucleus, Inferior Nucleus, etc.). Sparse representations with minimum overlap could be considered, according to Barlow [4]. Griffiths and Warren [5] propose that auditory object representations might be segmented or segregated in the Planum Temporal (PT) by increasing the independence between the neural activities. We therefore explore the potential of a hierarchical neural assembly - with the use of a computer simulation - whose layers increase the feature independence during training, to represent auditory object parts. It is observed that learned features are organized into non-overlapping maps (Figure 1) and that redundancy of the representation is in fact reduced. Learning was done on three categories of sounds having distinct acoustical statistics: speech, music and natural sounds. We observed that the learned feature maps are very different from one sound category to another and might be, to some extend, comparable to receptive fields measured in the brain. We discuss of their potential similarity with receptive fields measured in the Inferior Colliculus of the Guinea Pig and how they might be part of a representation of auditory objects in the brain.
